# Preadolescent children’s perception of power imbalance in bullying: A thematic analysis

**DOI:** 10.1371/journal.pone.0211124

**Published:** 2019-03-08

**Authors:** Helen J. Nelson, Sharyn K. Burns, Garth E. Kendall, Kimberly A. Schonert-Reichl

**Affiliations:** 1 School of Nursing, Midwifery and Paramedicine, Curtin University, Perth, Western Australia, Australia; 2 School of Public Health and Collaboration for Evidence, Research and Impact in Public Health, Curtin University, Perth, Western Australia, Australia; 3 Educational and Counselling Psychology, and Special Education, University of British Columbia, Vancouver, British Columbia, Canada; 4 Human Early Learning Partnership, School of Population and Public Health, Faculty of Medicine, University of British Columbia, Vancouver, British Columbia, Canada; Boys Town National Research Hospital, UNITED STATES

## Abstract

Bullying in schools is associated with an extensive public health burden. Bullying is intentional and goal oriented aggressive behavior in which the perpetrator exploits an imbalance of power to repeatedly dominate the victim. To differentiate bullying from aggressive behavior, assessment must include a valid measure of power imbalance as perceived by the victim. And yet, to date, there remains no agreement as to how to most accurately measure power imbalance among preadolescent children. This qualitative study explored children’s (age 9 to 11) understanding of power imbalance through thematic analysis of focus group discussions. Subthemes that emerged as influencing power imbalance include: age of victim, peer valued characteristics, and group membership and position. Subthemes of empathy and peer valued characteristics emerged as protecting against the negative impact of power imbalance.

## Introduction

The public health burden associated with bullying in schools has resulted in extensive research efforts toward understanding why bullying occurs and how best to mitigate the deleterious effects of bullying on child health and well-being [1]. Bullying is defined as aggressive behavior that is *repeated* and in which the perpetrator, for his or her own benefit, exploits an *imbalance of power* to dominate the victim [2]. Bullying is strategic and goal oriented behavior that can result in physical or social harm to the victim [3]. The targeted child is likely to feel less hope of a successful resolution when the perpetrator is perceived as more powerful, this in turn increases harm to the victim [4]. In comparison to students who are victimized without power imbalance, those who report frequent victimization with perceived power imbalance are more likely to experience: hopelessness, helplessness, interference with school work, and a loss of perceived support [5]; higher threat and lower perceived control [6]; lower life satisfaction and poorer school connectedness [4], higher risk of anxiety, depression and low self-esteem [7]. For this reason the context of power imbalance is central to bullying research [8].

At the age of 9 to 11 years children are increasingly likely to be involved in bullying behavior as they place more and more value on the peer group [9]. At this age higher order cognitive processing in the prefrontal cortex is emerging [10]. Children of this age have a growing capacity for self-reflection and increasingly make social comparisons, placing worth on qualities that are valued by peers and on belonging within the peer group [11]. Peer rejection becomes increasingly salient to the formation of identity [12]. For this reason children in this age group are an important target group in the development and implementation of school-based bullying interventions. The accurate measurement of bullying, however, is an ongoing issue; in particular repetition and power imbalance are not consistently acknowledged or measured by children’s self-report, often resulting in the incorrect reporting of aggressive behavior as bullying [8]. This has contributed to difficulty in evaluating school-based interventions and in comparing prevalence rates when different measures are used [5].

Child self-report is frequently used to obtain reports of victimization because the child’s own perspective is crucial in determining the bullying behavior [2]. Self-report surveys of bullying behavior typically begin with a definition of bullying that is intended to help children differentiate between aggression and bullying [2]. This has proved to be problematic when children either do not read the statement or they do not understand it. For example, in a survey of 19 children (aged 11–15) who identified themselves as victims of bullying by a written definition, only 10 were confirmed to meet the criteria at interview [13]. Of the remaining nine children, eight did not understand the definition and apply it appropriately. Furthermore, it has been shown that children’s own definition of bullying is different to that of researchers in that it rarely includes repetition and power imbalance [14]. It is possible therefore, that children who do not read or comprehend a definition will not differentiate aggressive behavior from bullying. In an attempt to overcome this problem, some researchers have used individual survey questions to assess children’s report of power imbalance [4,6].

The Californian Bully Victim Scale (CBVS) measured power imbalance in terms of *physical strength*, *popularity*, and *smartness in schoolwork* [4]. Reported test-retest stability was high. Green and colleagues [15] introduced items to the CBVS to measure power differential: how *likeable*, *good looking*, *athletic*, *old*, and how *much money* the perpetrator had in comparison to the respondent. Item reliability was not reported. Power imbalance was not associated between the CBVS and the definition based prevalence item of the Olweus Bully/Victim Questionnaire (OBQ) [16]. The association with psychological symptoms was strongest for the OBQ. Based on their finding of only fair agreement between measures Green et al. [15] proposed that the OBQ item measured repeated victimization rather than the presence of power imbalance. This is in contrast to suggestions that power differential contributes to repetition and harm [8]. Hunter et al. [6] found that *group size* and *physical size* were important aspects of power imbalance for boys whereas *popularity* and *physical size* were important for girls. Further research into the measurement of power imbalance at item level by self-report has been recommended, specifically to understand the behaviors that students engage in within the ecological context of the school environment [7,8].

Furthermore, Olweus [2] has proposed that perceived power imbalance might also relate to differences in status in the peer group. Consistent with this view, in a qualitative study that involved one-on-one interviews with 12 year old children, social position in the peer group was found to include an element of power, with the majority of participants identifying bullying as a group process [17]. Taken together, these studies underline the importance of obtaining children’s perspectives on bullying as a way to shed light on the ways in which children perceive bullying. Such information would not only advance the science and theoretical understanding of bullying, this information will also provide practical information for the design of effective measurement strategies and effective practices in schools to curb bullying.

### Research framework

The research framework of relational developmental systems theories emphasizes that developmental outcomes occur in response to the interaction between children and their environment [18]. Within these theories, which are based on Bronfenbrenner’s bioecological model, the *environment* includes influences ranging from children’s nearest relationships to political and historical influences. These affect the material and psychological resources available to children, shaping the feelings that children experience, for example threat, doubt, comfort, or hope, and associated neurobiological development over the lifetime [19,20]. Key to relational developmental systems theories is the concept of resilience, which is shown in adversity [21]. It is anticipated that this research, which is based on the framework of relational developmental systems theories, will increase our ability to promote resilience, as we understand the experience of students who struggle to escape the cycle of bullying victimization.

Within this framework, the integrity of measurement is supported by using qualitative research to inform instrument design within a culturally specific context, supporting research validity, transparency and clarity [18]. This paper reports the qualitative findings of a study that engaged children, the experiential experts of school bullying, in focus groups to identify factors related to power imbalance experienced at school in the context of aggressive acts between peers, some of which might qualify as bullying. This study contributes to the school violence literature by informing the capture of variables of power that are relevant to the study population based on qualitative analysis. In addition to the forms of power reported in this paper, students spoke of power differential as aggression that is intentionally hidden from adults in the school environment, reported elsewhere [22].

## Materials and methods

Focus group data were obtained and thematic analysis was undertaken to explore preadolescent children’s perception of power imbalance in bullying. Thematic coding built on existing knowledge, grouping data that had meaning based on the literature and on new ideas identified through focus groups [23]. Ethics approval was obtained from the Curtin University Human Research Ethics Committee (RDHS-38-15) and the Principal of the participating School.

### Sample

Focus groups comprised a purposive sample of children enrolled in grades 4 to 6 (ages 9 to 11) at one low fee paying private school in the Perth metropolitan region of Western Australia in July 2015. Consistent with nearby public primary schools, the school was placed within one standard deviation above the mean of socio-educational advantage in Australia [24]. The school has a dress code in which all children wear a formal uniform and a policy of no tolerance for physical or verbal bullying. The principal reported that, for this reason, it is more likely that bullying perpetration within the school would be subtle and relational. Subtle forms of bullying have been recognized as an issue in the context of similar school environments in Australia [25]. The principal was asked to purposively select children who would have the understanding and confidence to participate in a group without dominating or being intimidated [26]. A total of 30 children were invited to participate in three focus groups, one for each grade. Active consent was received from parents/guardians for 22 participants (73.3%), two parents/guardians declined consent and six forms were not returned. Two children were absent from school on the day of data collection. Children gave written assent for their own participation and for the focus group discussion to be audiotaped and transcribed.

The grade 4 focus group comprised 5 girls and 2 boys, the grade 5 group 4 girls and 3 boys, and the grade 6 group 3 girls and 3 boys. Parents identified their children as Australian (*n* = 17) and British (*n* = 1) (ethnicity was not available for two children). One family identified that a language other than English was regularly spoken at home.

### Focus group procedure

Focus groups were conducted in the reading room of the school library; the facilitators were experienced in working with children of this age [26]. Children were able to withdraw from the research at any time without negative consequence with provision made for immediate care by the school psychologist or chaplain for any child who became distressed by the discussion. Support was not required and all children participated for the duration of the focus group. Focus group questions were informed by the literature [27]. The focus group discussion guide was tested with three children from the age group prior to administration. No changes were made to the discussion guide. Consistent with other bullying research [17] a vignette was developed to introduce children to the topic of power imbalance. The vignette was based on social exclusion, a common behavior for this age group, and popularity, a key concept in relation to bullying at the group level [9]. The name Jordan is a common name for boys and girls in Western Australia and was chosen for the perpetrator to make the scenario relevant for the mixed gender focus groups. Olivia was chosen for the victim consistent with research suggesting that relational aggression is a salient issue to girls at preadolescence [4].

Olivia arrived at school one day and the children that she normally sat with and played with were talking about her and laughing at her. When she asked why, she found out that someone who she had thought was a friend had told a lie about her and now the other children did not want to include her. The kid that told the lie was named Jordan, and Jordan was very popular with the other kids.

The vignette was used to prompt third person discussion, which then lead to discussion about the types of bullying behaviors and related issues that happened at their school for boys and girls. Questions included “Tell us how you think Olivia might be feeling?” “Why do you think Jordan did this to Olivia?” “Can you tell us what do you think bullying is?” “Do you think some kids are more likely to get bullied/bully than others? Tell us about them” [22]. In addition, children were invited to make their own reflections about bullying. Focus group discussions were recorded using two audio recorders and one facilitator made a written observation of the non-verbal behaviors of children. Data collection ended when no new patterns emerged from the discussions. The focus groups lasted for 48 minutes (grade 4), 45 minutes (grade 5) and 50 minutes (grade 6). The software package NVivo 10 for Mac was used for data management and a research diary was kept.

### Data analysis

The first author transcribed these data verbatim. To achieve credibility, planned and systematic steps of thematic analysis were followed to ensure children’s views were represented (see Table 1). Rigor of the research was supported by the rich data generated from a purposive sample and collected in an environment familiar to children.

**Table 1:** Steps of thematic analysis.

Steps	Description of the process
1. Data collection	Focus group facilitators looked for patterns of meaning and interest during discussion, following up on comments made by children to explore meanings. Non-verbal communication was documented [26].
2. Become familiar with the data	Data were transcribed verbatim, read and re-read, and notes taken of initial ideas. Transcriptions were reviewed by the first and second authors to maintain dependability and determine credibility [28].
3. Initial codes	Codes refer to the systematic grouping of the most basic elements of the raw data that have meaning based on the literature. The raw data was grouped into codes based on new ideas identified from focus group discussion and from repeated patterns across the data set that had meaning based on the literature. Specific attention was given to children’s perception of power imbalance, coding as many themes as possible while maintaining tensions and inconsistencies within the data.
4. Generate initial themes	Relationships between codes and themes were explored and initial codes that did not sit into main themes were discarded or set aside for later review [29]. Themes were identified from the analysis of the data rather than from focus group questions.
5. Review and refine themes	Confirmation that the data supported each theme around a central concept. Rereading the data set to code missed data and to ensure that themes accurately reflected the meanings relayed by children. A thematic map was built.
6. Define and name themes	The meaning captured by each theme was organized into a narrative identifying what was of interest, why, and how it fit into the overall picture in relation to the research question. Sub-themes within themes demonstrated subsets of meaning within the data. Themes were named.
7. Write the report	The story of data was written to show the merit and validity of the analysis. The plausibility of the argument was explained based on the literature, focus group data, and theoretical framework.

The steps of thematic analysis are based on Braun and Clark [23]

## Results

Results present the major themes identified by thematic analysis of focus group discussions: 1) influencing power imbalance, defined as factors that are influential in increasing the power imbalance experienced by children who are bullied. 2) Protecting against the negative impact of power imbalance, defined as factors that buffer against bullying.

**Fig 1:**
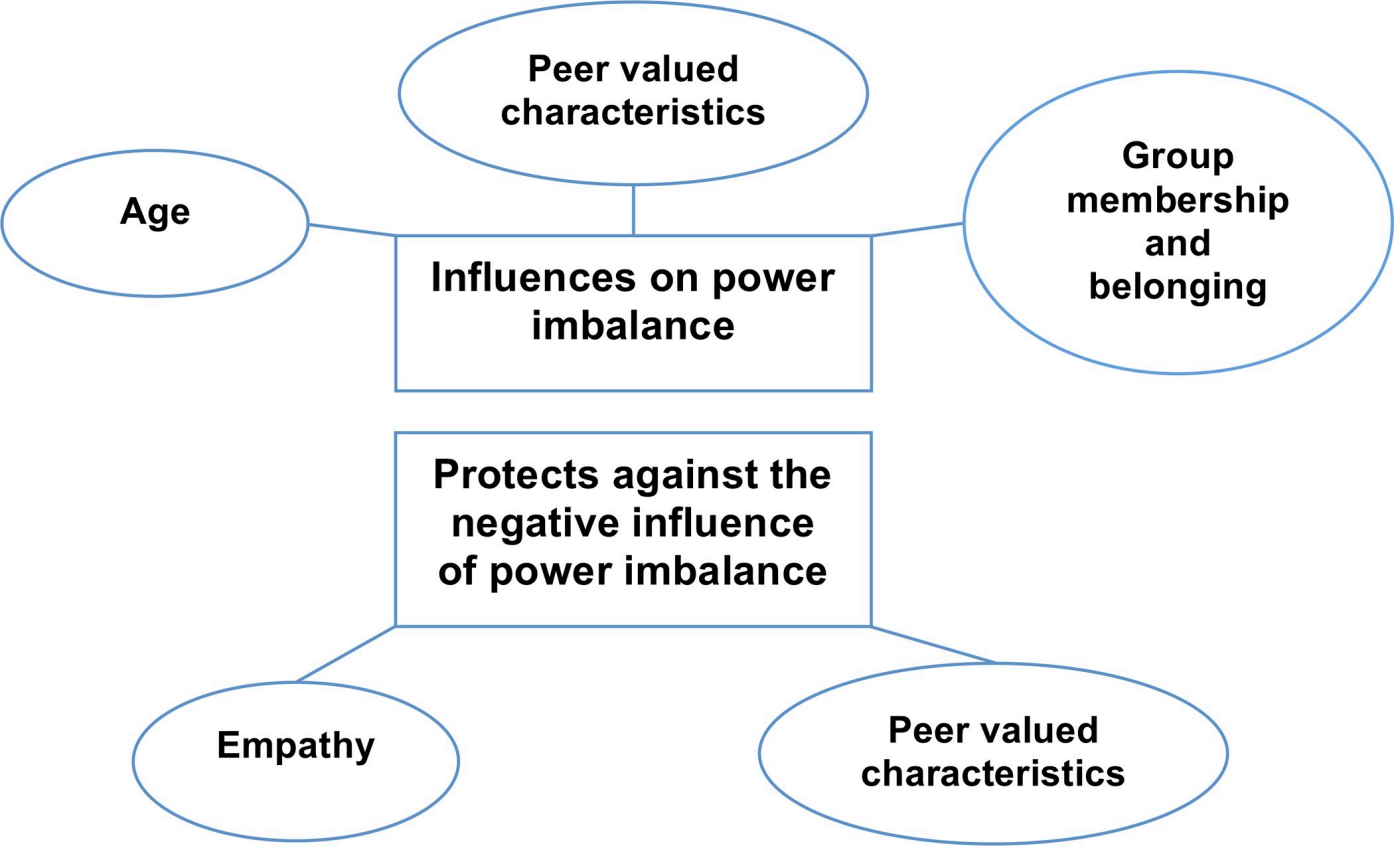
Thematic map of factors that influence and protect against power imbalance. Two major themes of the thematic analysis emerged: 1) influences on power imbalance, and 2) protects against power imbalance. Subthemes of *age*, *peer valued characteristics*, and *group membership and belonging* were identified as influencing power imbalance. Subthemes of *peer valued characteristics*, and *empathy* were identified as protecting against the negative impact of power imbalance. This figure is adapted from [22].

Subthemes identified as influencing power imbalance were: age, peer valued characteristics, and group membership and position. Subthemes of peer valued characteristics and empathy emerged as protecting against power imbalance (Fig 1). Comments that support each subtheme have been referenced using pseudonyms (children’s real names are not used), grade at school, and gender.

### Major theme one: Influences on power imbalance

#### Sub-theme one: Age

Three children perceived that age represented a form of power imbalance. Walter (grade 5 boy) said, “I personally think the common type of bullying is an older kid is bullying a younger kid cause they think um they’re better since, um, they’re elder than them.” Kailey (grade 4 girl) recounted a story remembered from grade 2 of a student who involved “all her friends and all the grade 6 friends” to intimidate Kailey. Gayle also highlights that older children are often influential in the school environment, which might impact the protective factor friends provide.

Somebody starts a rumor and then girls tell girls and girls tell girls and from grade 4 it goes all the way to grade 6 and then you, you have friends and then they’re not your friends and they won’t let you sit with them. (Gayle, grade 4 girl)

#### Sub-theme two: Peer valued characteristics

Lucy provided a firm response to the question "are some kids more likely to be the bully than other kids?"

People who are really smart and pretty and who are really popular, so they just bully the people who aren’t because they are people who are easy to get. (Lucy, grade 4 girl)

Lucy talked of the people who are “easy to get” in the context of social dominance, the dominance resulting from peer valued characteristics. In each focus group *appearance*, *sport*, and *smart* occurred as subthemes of peer valued characteristics.

Almost all children identified *appearance* as a reason for being bullied. Daisy (grade 4 girl) said that children are bullied for “how they look”, and Maria (grade 5 girl) said, “They might bully that person because they don’t look right.” Hope (grade 6 girl) said that children “judge” others on “what they look like”. A few children suggested that students might also be bullied because they are popular and attractive. Vashti (grade 5 girl) gave conflicting opinions, referring to children being bullied “because they think ‘oh, you’re out of shape’ like, oh you’re ugly”, but also defending popular children saying they are bullied because they are pretty. Vashti’s defense of popular children was in response to an observation by Grace (grade 5 girl) that popular children are more likely than others to bully others.

Kailey (grade 4 girl) suggested that “Not being the same as everyone else and not having like what everyone else has” influenced being bullied. Luke (grade 5 boy) gave an example of needing the most up-to-date mobile phone and Grace (grade 5 girl) spoke of electronic and online games: “They’ll say, ‘oh that’s a different game, that's for losers’, and they’ll start laughing at you.”

Children from all three focus groups suggested that children who were good at *sport* were less likely to be bullied, however bullying could also occur on the sports field. Stefan (grade 4 boy) referred to the bully as one who tries to “rule the game”, speaking of games such as football. Antony (grade 4 boy) referred to “physical” bullying on the sport field, “They like go up to you and grab you by the t-shirt and punch you.” This was discussed in the context of using the game as an opportunity to bully as opposed to aggression, which might be as a result of the game itself. Grade 5 children discussed social exclusion based on online gaming, however George (grade 5 boy) also discussed social exclusion in association with “skills like your soccer skills and basketball skills.” Grade 6 children also recounted stories of being teased about their athletic ability.

Talent was, however, not consistently related to a position of power. For example, Stefan (grade 4 boy) said that “talented” children were more likely to be bullied, attributing the bullying to jealousy as highlighted by Antony (grade 4 boy), “The ones that can’t do as much and the ones that think they’re not good, they most likely bully the ones that are good.”

Lucy (grade 4 girl) said, “people who are more likely to bully other people are people who are really smart.” Grade 6 children similarly spoke of power imbalance in terms of high academic achievement. Ella (grade 6 girl) said “I also think like if someone’s if not like as good as a subject as you are, doesn’t mean you put them down.” When discussing what type of children are more likely to bully others and to be bullied, children from all three focus groups suggested that children who were *smart* had the skills to get away with bullying. For example, Luke (grade 5 boy) said that the teacher wouldn’t expect smart children to be the bully and George (grade 5 boy) related being smart to getting “their way out of trouble when (sic) the bullied kid tells on them.”

#### Sub-theme three: Group membership and position

The group process of bullying was the third major subtheme identified in relation to power imbalance. Within this subtheme, children referred to the power of the *big bully*, to *group membership*, and to *cyber bullying*.

When discussing the group aspect of bullying children in each grade referred to one main bully, named by Antony (grade 4 boy) and Walter (grade 5 boy) as “the *big bully*”, referring to the leader of the group as opposed to big in size. The leader of the group holds a position of power and has control over who is or is not accepted within the group as shown by Gayle (grade 4 girl), “And that girl that was bullying me never let this one girl in grade 2 play with us because she, her hair was always pretty scruffy.” Grace, and Ruby discussed how bullying occurred to secure power within the group, either through bullying other children or bullying those within the group.

I think that sometimes the really popular people. They bully the normal people um to (show) they’re the best, “I can do whatever I want and if I’m popular I can do what, a lot of things that I want and I want to tease you because I’m the best.” (Grace, grade 5 girl)

Um, my, friend, she was getting really angry with me … because I wasn’t telling her something about somebody else had told me not to tell anybody … and then her whole group started ganging up on me and telling me like “oh, you should tell her”, or “tell me, I won’t tell anybody”. (Ruby, grade 6 girl)

Gayle left the group to support the child who had been bullied and Ruby similarly “made another group of friends”, removing herself from the negative group dynamic. In doing so both girls showed empathy and a development of self. Conversely, Hope (grade 6 girl) spoke of children’s fear of exclusion, highlighting how the need to remain in the friendship group further enhances the power of the group leader: “Just too scared (to stand up for themselves) because they don’t want to be excluded from their friends.” Vashti gave a similar example, supporting the discussion of focus groups, which suggested that some children remain in friendship groups despite being the target of aggression.

Sometimes a person is like really, really popular and a person’s like hiding in the shadows, kind of something weird, and they’re just like oh, I want to be like them, so they’re like trying to be friends and the person who’s popular is actually really nice and they’re just like “oh do this and that” and then when they do like become best friends like a really good connection the faker, the one, the person who wanted to be like them, just tells some rumors to get that person down the bottom of the popular list and they just say “oh, get it, I’m the most popular person here”. (Vashti, grade 5 girl)

In an earlier discussion Vashti said “sometimes, people think that, like when they’re the bully, they think that if we scare people those people will be nice to us”, suggesting that Vashti was engaged in a struggle for social dominance, potentially as both aggressor and victim. Similarly, grade 6 children referred to bullying as being motivated by a goal of social dominance. Carlton (grade 6 boy) stated that bullying is "Just a way that they try to win and be the top, and be on the top.” The leader holds control over the group through fear, as stated by Gayle (grade 4 girl), “I think everybody takes the bully’s side because they’re scared of being bullied.”

Grade 6 children also focused on the perceived value of *group membership*, for example belonging to the popular group. Hope (grade 6 girl) said that bullying makes others feel as if they don’t belong and “like they need to change to be with other people at school so that they’re not lonely”. Ruby (grade 6 girl) gave an example of a girl who responded to gossip by trying to "change to be just like them". Roland (grade 6 boy) talked of children changing “everything”, “their entire personality. Their clothes, their feelings… just to fit in.”

The focus group discussion highlighted that bullying often happens in a group with group members playing different roles. The pressure comes from within the group but possibly also in support of one main protagonist. However other group members also play important roles. For example, Walter also highlights the role of the bystander.

Like this group of kids just comes into the toilet and some kids just block the um, block the door and then, then no one sees, and then, like they start um, and the person inside the um, the bullier (sic), starts bullying the um, kid. (Walter, grade 5 boy)

Children in the grade 5 and 6 focus groups referred to *cyber bullying* or online bullying, whereas grade 4 children said that they tended not to have access to online forums. Grade 4 children were therefore unlikely to experience cyber bullying. The focus on cyber bullying included discussion about social media and online games. Grace (grade 5 girl) spoke about the group effect of bullying through social media, “Um, telling lies about people and talking about them behind their backs to people, and like also that also happens on the internet with your friends.” Hope (grade 6 girl) gave a similar example, “They say some really rude stuff, and then it kinda gets bigger and bigger, and then more people get dragged in, like Instagram.”

Luke (grade 5 boy) spoke of “Online gaming like Minecraft and all those games, sometimes that (cyber bullying) happens.” Walter (grade 5 boy) framed online gaming in terms of bullying within or by a group, “He gets bullied um, by someone in Clash of Clans in his own team.” While Walter was talking about the online games Vashti indicated shhh putting her finger to her mouth and looking directly at Edith. Edith later moved away from Vashti and sat closer to the boys. This might have indicated an attempt to control the conversation by Vashti.

### Major theme two: Protects against the negative impact of power imbalance

#### Sub-theme one: Empathy

Children in the grade 6 focus group referred to the importance of accepting the uniqueness of each person, within this the protective factor of empathy was evident. Hope (grade 6 girl) said that children who bully others “don’t understand what (sic), how other people are”. In response to the vignette Carlton (grade 6 boy) said that he would want to help Jordan “understand how Olivia feels right now because you’ve gone and told a lie to everyone. She doesn’t feel that good”. This observation reflected empathy. On the other hand, the following reflections by George and Ella show the callousness of bullying and associated lack of empathy:

I think the worst type of bullying is when let’s say, um say someone’s um mum died, and … the bully’s like “oh, your mum died, hahaha” like that, I think that’s the worst type of bullying because you could hurt, basically, their feelings a lot. (George, grade 5 boy)

They might like, put one of their friends under the bus, so they like might tell one of their friends like most valuable secret to the popular group and that might like just get them in. (Ella, grade 6 girl)

#### Sub-theme two: Peer valued characteristics

Having fashionable clothing and the latest technology (e.g. phone and electronic games) were seen to be important protective factors against being bullied for both boys and girls across all focus groups. This included “shoes” among boys and “their outfits” among the girls. Arthur (grade 6 boy) thought it was “unlikely” that good-looking children would be bullied and spoke of the protective factor of having “cool” clothes and “looks.” Although the protection of these characteristics was spoken of across each focus group, conversation was framed around the harm that was experienced by children who did not have peer valued attributes or belongings. As previously stated, another protection against power imbalance was to be “good at sport,” or to be “smart.” Gayle (grade 4 girl) suggested that children who were *smart* had the skills to stand up for being bullied, “Because they always have good comebacks.”

## Discussion

This research focused on identifying factors that influence power imbalance associated with bullying at preadolescence in the context of a middle class population in Perth, Western Australia. Age, peer valued characteristics, and group membership and position were identified as subthemes of factors that influence power imbalance. These are discussed beginning with age.

Grade 6 is the final grade of elementary school in Western Australia, and children from grades 4 and 5 spoke of intimidation from children of an older age group. This is an important consideration in research design; age difference across school grades represents a form of power imbalance that will not be captured by peer reports of bullying by a classroom roster or grade group [2]. The self-report of power imbalance by the victim is therefore important to the integrity of bullying research. Consistent with Green et al. [15] our focus group findings support *‘older than me’* as a measure of power imbalance.

Most children indicated appearance and athleticism as sources of power imbalance associated with bullying. At preadolescence children rely on peers for social comparisons and keeping up with the accepted norms, appearance and clothing become important to goals of acceptance and status [30]. Status places a buffer around popular aggressive children and increases power because peers value the privilege, identity, and resources associated with belonging in a group with others of social status [30]. Children in our research indicated that appearance includes fashionable clothing and belongings, including shoes and smart phones. Athletic skills were considered particularly important, this is consistent with a recent qualitative study in which teachers associated poor athletic ability with peer victimization [31]. Consistent with Green et al. [15], our focus groups support the addition of peer-valued characteristics *‘good looking’*, and *‘good at sport’* into a self-report measure of perceived power imbalance.

‘Smart’ was identified as a subtheme of peer valued characteristics from each focus group. Felix and colleagues [4] questioned the use of ‘smart’ as a measure of power imbalance, finding that only a few students identified “smart in schoolwork” as the only source of power imbalance (p. 240), this item was however maintained in the CBVS [7,15]. In our research children identified the word smart with high academic achievement, and additionally related being smart to being deceptive and avoiding any association with the bullying. This is consistent with research that suggests some teachers fail to recognize bullying posed by students who hold high social status [31] and that popular students, some of whom were school prefects, bully others [32]. While qualitative research found some children justified bullying others who were academically smart, termed a “nerd” [32], this research suggests that bullied smart children are more likely to have the skills to negotiate the situation. Thus, the use of the word ‘smart’ in relation to power imbalance is likely to be multifaceted. Children have a right to be heard [33], and children from each focus group referred to smart children bullying others. This supports the inclusion of *‘really smart’* into a self-report measure of power imbalance, allowing the a priori factor structure to be empirically assessed.

Popularity was repeatedly referred to as a goal of children who bully during the grade 5 and 6 focus groups. This could be a result of the reference to popularity in the vignette, however popularity is recognized as an appropriate measure of social dominance associated with bullying [9]. Table 2 documents individual items that have been used to assess power imbalance, the item *more popular* is a consistent measure of power imbalance in each previously cited study. In contrast, focus group participants spoke of children being aggressive toward others with the goal of increasing their own status while aiming to minimize the status of more popular children. Victimized children experienced a consequent feeling of hurt or experience of harm. Children who use the bistrategic behaviors of aggressive coercion and prosocial skills to achieve a goal of social dominance are popular with peers, in part because their social power is a resource [3]. Bistrategic children tend to selectively target high status children from their own social network as victims of relational aggression. It is however, unclear if victimization in this context represents a power differential and further investigation is recommended to inform interventions specific to this target group [3]. Thematic analysis of focus group discussion supported the addition of *‘trying to be more popular’* to a measure of power imbalance.

**Table 2:** Individual items that have been used to assess power imbalance.

Hunter et al. [6]	Felix et al. [4]	Green et al. [15]	Malecki et al. [7]	Focus groups (*N* = 20)
Physically stronger	Physically strong	Physically strong	Stronger	Much stronger than you^a^
In bigger groups				With a group of students^b^
More popular	Popular	Popular	More popular	Trying to be more popular
	Smart in schoolwork	Smart in school	Smarter	Really smart
		Good looking		Good looking
		Likeable		
		Athletic		Good at sport
		How much money		
		How old		Older than you
				In the most popular group
				Bigger than you^a^

^a^These items were added by students in the second round of focus groups (face validity).

^b^A review of identified themes supported the addition of this item following expert review.

The power associated with belonging to a group is another subtheme identified from thematic analysis. Hunter et al. [6] measured power imbalance by asking if the aggressor(s) was “in bigger groups”. The context of our research was a middle class fee-paying school in which a strong stance is taken against physical aggression. In this context bullying is likely to be perpetrated through the social dynamic of the group, with power exerted by the leader of the group, and through identity as a member of the group [3,17]. Members of the ‘in-group’ assist in the perpetration of bullying and defend others in the group to confirm a sense of belonging or as a result of peer pressure [9]. Thematic analysis supported the addition of *‘in the most popular group’* as measure of perceived power differential. Because this might reflect the focus of the vignette and following recommendation by an expert reviewer an additional item *‘with a group of students’* was included, consistent with the theme of group membership and position.

Hunter et al. [6] were the first to measure power differential using individual items; because no prior research had examined the effects of power imbalance the authors selected items to reflect potentially important types of power (see Table 2). Felix et al. [4] created the CBVS and the clarity and wording of each item was assessed in focus groups and Green et al. (2014) added items focusing on power to the CBVS. Our research adds to the current research by identifying items through thematic analysis of focus groups discussion, recognizing children as the experts of their own experience. Consistent with Hunter et al. [6], children placed importance on the function of the group in relation to power imbalance, however, rather than group size the focus was on *group membership and position*. Consistent with the CBVS, *age* and *peer valued characteristics* were identified as influencing power imbalance. *Empathy* was identified as protecting against the misuse of power (Fig 1).

### Factors that protect against power imbalance

Within our research, empathy was identified as a factor that may protect against power imbalance in peer relationships at preadolescence. Empathy contributes to moral and social development as emotions are aroused in children, including emotions of guilt [34]. Conversely, children justify their bullying or bystander behavior rather than acknowledging emotions of guilt or shame, motivated by a desire to belong in the group. This is associated with moral disengagement and a lack of empathy and is shown in the callousness associated with bullying [34]. Empathy is supported by the school context of adult and peer support as children learn to negotiate relationship stress [31]. For example, Ruby who experienced bullying at her previous school, and chose not to give in to the demands of others in her group when doing so would have caused harm to a friend. This highlights the importance of comprehensive and integrated whole-school interventions that aim to build a culture of belonging and in which self-blame is alleviated for children who are victims of bullying [31].

Within our research a tension was identified in the influence of peer valued characteristic’s on power imbalance. Items that have previously been attributed to power imbalance include being smart, good at sport or good looking [4,15]. In our study, thematic analysis has highlighted that characteristics including appearance, smart and athleticism can both influence and protect against power imbalance. In a recent review Volk and colleagues [8] commented on the complexity of bullying behavior, and recommended that qualitative research might help reveal different degrees of power within relationships. This gives insight into one of the complexities that underlies the measurement of power imbalance [35].

### Future directions

Bullying is complex and often hidden from those in authority, it is important to understand the social dynamics of the behavior from the perspective of children themselves, and within the cultural context, to assess causes, evaluate interventions, and implement policies [8]. There is, however, ongoing debate about how to measure the power difference of bullying in ways that are meaningful and that will inform the development of interventions [8,36]. This study is the first stage of a mixed methods study; the qualitative research has informed the design of individual survey items to measure children’s experience of power imbalance. As recommended by Tolan and Deutsch [37] different quantitative methods will follow, it is anticipated that a multiple method study will provide a framework by which to inform the understanding of measurement issues. Each item will be included in an online questionnaire and displayed when children report frequent victimization. The item set will follow the stem-question “When these things happened, was the mean student …” The quantitative phases of the research will employ exploratory and confirmatory analysis. It is anticipated that this will provide additional assessment of the meaningfulness of the survey items [36]. In addition, the resulting survey will be tested for invariance across different contexts, for example, different age ranges or different schools [36]. To our knowledge this is unique in bullying research.

### Limitations

A limitation of this research is the use of focus group discussion rather that one-on-one interviews, which would permit the exploration of some topics in more depth. However, the focus group environment facilitated our awareness of power dynamic as children responded to each other, a relational aspect of focus group discussion that would not be apparent in interview [38]. The small focus group sizes can be considered a limitation, however group size of four to eight is recommended for children and rich data may be generated from a small group [26]. A second limitation of the research is that the reference to popularity in the vignette might have biased children’s discussion; two additional items were recommended by students who subsequently assessed the face validity of the new instrument: *much stronger than you*, and *bigger than you*. However, the vignette was based on extensive review of the literature, and it can be argued that knowledge is lost by ignoring established findings [39]. The vignette also enabled good discussion in the third person. Third, the mixed gender of the focus groups could be considered a limitation however, there is not universal agreement on this [26]. The non-verbal interaction between participants in the grade 5 focus group suggested that the power dynamic was not restricted to relationships that were exclusively between girls or boys, supporting mixed gender focus group composition. A fourth limitation is that our research focuses on children’s experience of power imbalance within the specific context of urban middle class Australia, representing a potential bias in sample selection. However, within the framework of developmental systems theories, development is studied within the unique ecological conditions that contribute to individual outcomes. The focus on the middle class was consistent with many public and private schools in the metropolitan region of Perth, Western Australia, supporting the context specific validity of instrument design. This gives strength to the research. A fifth limitation is that participants’ experience with offending, victimization, or both were not controlled for, however it is recognized that most school students have some experience with bullying situations, either directly or as an active or passive bystander [40].

## Conclusion

Bullying is complex and often hidden from those in authority, it is important to understand the social dynamics of the behavior from the perspective of children themselves, and within the cultural context, to assess causes, evaluate interventions, and implement policies [8]. This study used qualitative analysis to inform the context specific understanding of power imbalance in schools in which a strong stance is taken against physical bullying. Researchers have previously used individual items to measure power imbalance, and have found that items such as “smart” might not be an adequate measure of the power imbalance that is experienced by children who are bullied. In contrast, children in focus groups suggested that peer-valued characteristics including smart, appearance, and being good at sport either influence power imbalance or act as a buffer against bullying, protecting against power imbalance. This finding gives insight into one of the complexities associated with measuring power imbalance.
